# Leadership and the functioning of maternal health services in two rural district hospitals in South Africa

**DOI:** 10.1093/heapol/czx174

**Published:** 2018-07-08

**Authors:** T Mathole, M Lembani, D Jackson, C Zarowsky, L Bijlmakers, D Sanders

**Affiliations:** 1 University of the Western Cape, Robert Sobukwe Road, Bellville, South Africa; 2 University of Montreal Hospital Research Centre (CR-CHUM), 850, rue St-Denis, Montreal (Québec) Canada; 3 Radboud University Medical Centre, Radboud Institute for Health Sciences (RIHS), Geert Grooteplein-Noord 21 EZ Nijmegen, The Netherlands and; 4 Department of Social and Preventive Medicine, School of Public Health, University of Montreal 7101 av du Parc, Ste, Montreal, Québec H3N 1X9 Canada

**Keywords:** Leadership, maternal health, health systems, obstetric care, managers, South Africa, district hospital

## Abstract

Maternal mortality remains high in Eastern Cape Province, South Africa, despite over 90% of pregnant women utilizing maternal health services. A recent survey showed wide variation in performance among districts in the province. Heterogeneity was also found at the district level, where maternal health outcomes varied considerably among district hospitals. In ongoing research, leadership emerged as one of the key health systems factors affecting the performance of maternal health services at facility level. This article reports on a subsequent case study undertaken to examine leadership practices and the functioning of maternal health services in two resource-limited hospitals with disparate maternal health outcomes. An exploratory mixed-methods case study was undertaken with the two rural district hospitals as the units of analysis. The hospitals were purposively selected based on their maternal health outcomes: one reported good maternal health outcomes (pseudonym: Chisomo) and the other had poor outcomes (pseudonym: Tinyade). Comparative data were collected through a facility survey, non-participant observation of management and perinatal meetings, record reviews and interviews with hospital leadership, staff and patients to elicit information about leadership practices including supervision, communication and teamwork. Descriptive and thematic data analysis was undertaken. The two hospitals had similar infrastructure and equipment. Hospital managers at Chisomo used their innovation and entrepreneurial skills to improve quality of care, and leadership style was described as supportive, friendly, approachable but ‘firm’. They also undertook frequent and supportive supervisory meetings. Each department at Chisomo developed its own action plan and used data to monitor their actions. Good performers were acknowledged in group meetings. Staff in this facility were motivated and patients were happy about the quality of services. The situation was different at Tinyade hospital. Participants described the leadership style of their senior managers as authoritarian. Managers were rarely available in the office and did not hold regular meetings, leading to poor communication across teams and poor coordination to address resource constraints. This demotivated the staff. The differences in leadership style, structures, processes and work culture affected teamwork, managerial supervision and support. The study demonstrates how leadership styles and practices influence maternal health care services in resource limited hospitals. Supportive leadership manifested itself in the form of focused efforts to build teamwork, enhance entrepreneurship and in management systems that are geared to improving maternal care.

**Key Messages**

- This study demonstrated that strong and committed leadership can promote an effective mobilizsation of teams and creates the conditions of good performance in districts hospitals even when they have limited resources and support from higher level structures.
- In the poorly performing hospital, lack of approachable and effective leadership led to, low morale, frustration, lack of commitment and persistence of other confining and restrictive factors.
- This research reinforces similar findings in maternal health and other programme areas that, while the technocratic and financial issues (including guidelines and tools) are valuable and essential, their successful application will be determined by the context and, in particular, leadership capacity.

## Introduction

This article examines how leadership styles and practices influence the functioning of maternal health services using case studies of two district hospitals in a rural district in South Africa with disparate maternal health outcomes in a similar context. Tinyade Hospital (pseudonym), a poorly performing hospital, and Chisomo Hospital (pseudonym), a better performing hospital. In an earlier study of health systems and maternal health outcomes in this district, leadership had been identified as a key driver of performance in maternal health services (Lembani *et al.* 2015). This study sought to elucidate these preliminary findings in a particular context, and to contribute to the developing body of scholarship and practice aimed at better understanding leadership and how it improves or undermines health services functioning in low- and middle-income countries (LMICs).

### Leadership, health systems and the front lines

Leadership is broadly regarded as essential for effective health systems development (Gilson and Daire 2011), and it is one of the building blocks in the WHO health systems framework (Curry *et al.* 2012). It is described as the most influential factor in shaping organizational culture (Gilson and Daire 2011; West *et al.* 2015). Increasing calls to nurture effective leadership practices in health care draw on strong evidence of association between leadership and a number of systems outcomes, such as patient satisfaction, organizational financial performance, staff satisfaction and engagement as well as overall quality of care and health outcomes (Shipton *et al.* 2008; West *et al.* 2015). In management science, leadership is viewed as critical to the success of organizations (The Governance Institute 2015).

Despite the acknowledged importance of leadership in health care management, little research has been published on leadership experiences in LMICs especially in Sub-Saharan Africa (Curry *et al.* 2012). Recently, there has been growing interest in highlighting this aspect of health systems. Gilson (2016) explains ‘… the most complex challenge in health systems is to nurture persons who can develop the strategic vision, technical knowledge, political skills and ethical orientation to lead the complex processes of policy formulation and implementation because without leaders even the best designed systems will fail’ (Gilson 2016, p. 187).

Complementing typical understandings of management, which focus on ‘doing things right’ through improving efficiencies, the notion of leadership highlights ‘doing the right things’ by focusing on effectiveness (Gilson and Daire 2011; Walters 2016). Most leadership theories were established from the business perspective and only recently applied to public healthcare settings, which have different objectives and in which the nature of the work—literally ‘life and death’—poses different challenges to individual and collective action among both staff and managers (Al-Sawai 2013). Leadership in health care settings involves decision-making for complex systems comprising both the hardware (human resources, finances, medicines and technology, organizational structures, service infrastructure and information systems) and the software aspects (ideas, interests, interrelationships, trust, power, values and norms) (Sheikh *et al.* 2011). Kwamie (2015) cautions it is important to balance the functions of management and leadership as both are crucial for effective functioning of complex health systems, particularly at the level of translation of policies into action by managers and providers at the frontlines. Gilson (2016) discusses how frontline managers deal with the everyday politics and realities of policy implementation. The frontline managers are the connecting points of the organization to the higher management levels. Their leadership needs to create an enabling environment for ‘interactions and networking among actors that sustain collective action towards shared goals and support innovation and adaption’ (Gilson 2016, p. 190). This requires distributed leadership approaches that ensure collective action and teamwork.

Despite this acknowledgement of the need for effective leadership, the research to understand how leadership plays out at the level of health facilities and individual service providers is limited. This study was undertaken to address this gap. The study objectives were to document and analyse the leadership styles and practices and functioning of maternal health services in two resource limited hospitals with different maternal health outcomes.

Researchers use different indicators in evaluating leadership effectiveness, such as organizational outcomes known as objective indicators or ratings from peers, subordinates or superiors which are referred to as subjective indicators (Yukl 2013). Yukl discusses how difficult it is to measure leadership effectiveness and to decide which indicator or set of indicators is the most appropriate. In this article, we use both organizational outcomes and ratings from subordinates to compare leadership effectiveness in the two selected hospitals. This assessment of micro-contexts and processes is important to be able to understand and address the variability of outcomes in apparently similar circumstances. Walters (2016) have shown how even when the external contexts in which hospitals operate are similar in many respects, the way each hospital is able to function and respond to the chronic challenges and the levels of engagement and innovativeness may be very different, depending on its internal context (Walters 2016). In an earlier study on health systems and maternal health (Lembani *et al.* 2015), we found that the two selected hospitals functioned differently and our analysis of the major drivers of performance indicated that leadership was one of the underlying factors that contributed to the improvement of functionality of the maternal health services.

### Study context

South Africa has placed considerable emphasis in recent years on improving health management, including several strategies such as ‘Primary Health Care re-engineering’ and ‘the Ideal clinic’ (Gilson and Daire 2011). Despite these efforts, inadequate leadership capacity among managers has been identified as a key challenge constraining effective health service delivery (Wilson *et al.* 2015).

Eastern Cape Province is one of the poorest areas of South Africa with poor roads, long distances to hospitals and high transport costs, where maternal mortality remains high, despite over 90% of pregnant women utilizing maternal health services (Human Rights Watch 2014).

The study district is one of the worst performing districts in the country, with poorer health outcomes, such as high home delivery rates, HIV prevalence, teenage pregnancy rates, and HIV/TB co-infection rates. The district health system reports high vacancy rates of professional staff (between 30 and 70%), and it has old and poorly maintained infrastructure. In our 2013–14 assessment of the health system challenges that affect the performance of maternal health services in the study district, leadership emerged as one of the key challenges at the health facility and district level (Lembani *et al.* 2015). There was high staff turnover, even among senior managers, that affected continuity and stability of the health system. New senior managers were found to change policies and reshuffle senior staff. This was accompanied by poor resource management, with budgets being either overspent or underutilized, as well as corruption, and low accountability according to conversations with the province’s senior management. The aim of this study was to build on these initial findings to deepen understanding of how leadership affects the functioning of maternal health services through examining two district hospitals in the same region with similar health systems challenges, but with different health outcomes.

We therefore set out to examine each hospital as a separate case in order to gain a better understanding of the leadership phenomenon, and we therefore hope to gain insight into the complexity of the facilitating factors and the challenges faced in the local context of these hospitals. Our hypothesis was that strong, committed and supportive leadership enables a hospital to develop mechanisms that help them to cope with everyday health systems challenges in a poorly resourced context, and to positively influence the general performance of maternal health teams and services.

## Methods

A mixed-methods case-study design was used to draw multiple perspectives and explore the complexity and uniqueness of these cases within their contexts (Yin 1999; Dopson 2003). One successful and one less successful hospital based on variation in selected maternal health indicators obtained from the district health office between 2008 and 2012 were purposively selected from 11 Eastern Cape province district hospitals. Other selection criteria were that both hospitals were in the same district, with similar health systems challenges, size and case mix. Each hospital was treated as individual cases and analysed separately showing similarities and uniqueness of each case (Eisenhardt and Graebner 2007).

Data were collected through document analysis, non-participant observation and interviews. Initial activities included the review of records, programme documents, work plans and reports, as well as meeting minutes. Documents were sourced from the facilities, district health department and Non Governmental Organisations (NGOs). Information from this initial review provided context interpretation of results and refinement of data collection instruments. The second phase of data collection (October 2013–November 2015) included survey questionnaires, non-participant observations of management meetings, compilation of hospital data on case-loads and resources, staff interviews and discussions related to attitudes, teamwork, training, communication, supervision, managerial support and leadership.

Key staff members involved in health systems and maternal health care at district and facility level were invited to participate in the interviews to ensure that views of all stakeholders were taken into account. Inclusion of criterion for this group of participants were that participants had leadership roles or that they had been involved in maternal health activities for at least 2 years in the district or hospital. We interviewed the district two senior manager and maternal and child health coordinator; as well as nine staff members from the maternal and child health unit, two clinical managers, two facility managers and four representatives from the hospital boards from the two hospitals. Notes were recorded during discussions and observations made. Four management meetings and six perinatal meetings were observed and audio recorded after participant permission.

Semi-structured interviews were conducted with health care providers to explore experiences and perceptions of the quality of obstetric care, and management and leadership issues, including: orientation, training, supervision, performance management, communication, staff attitudes, teamwork and relationships.

Quantitative data were collected on human resources available in the two hospitals, as well as availability of equipment, infrastructure and drugs and supplies crucial for a functional maternal health unit. Modified Averting Maternal Death and Disability (AMDD) data collection tools were used (WHO 2009). Data were also collected from the district information system on patient headcount, facility deliveries, caesarean sections and patient complaints. Non-participant observation was used to document patient interactions with providers. Data were collected by a member of the research team with assistance from Research Assistants from the district, one of whom was a nurse by profession and another, a health promotion graduate. Interviews and discussions took 45–90 min and were audio-recorded and transcribed.

Qualitative data were analysed simultaneously through a systematic technique of thematic and constant comparative analysis. This involved an iterative process between interview results in producing a multifaceted description of the context, policy environment and experiences of health care professionals and leadership in each of the cases. A preliminary coding scheme used descriptive codes from transcribed data and notes. The thematic coding analysis was inductive and codes derived from the data. Initial codes were descriptive and close to the data (Thorne *et al.* 2004). This preliminary coding scheme was subsequently applied using atlas.ti software to relevant interview segments. Atlas.ti software was used to group similar codes and then organize data into categories. Finally, relationships between themes and responses of different participants were identified (Thorne *et al.* 2004) and themes refined through integrating data from multiple sources into a coherent whole (Toma 2000).

Quantitative data were analysed using SPSS. Descriptive analysis was performed to describe the distribution and range of responses to each variable. Response categories were constructed after coded responses were entered into the data base. Data collected from the district information system were analysed in Microsoft Excel. Data were triangulated to further enhance validity.

## Ethical considerations

Permission was obtained from the author’s institute, the Eastern Cape Provincial Research Committee, the district and the two hospitals. Prior to data collection, verbal and written informed consent, including permission to audio-record, was obtained from all participants. Participants were advised that they could refuse or withdraw any time without giving reason. There were no refusals or withdrawals.

## Results

Four key themes emerged from the analysis. The first theme relates to external context and infrastructure, including the history and formal structure of the two hospitals in the district health system, patient profiles, volume, case mix, human resources, working conditions, availability of emergency obstetric equipment and drugs. In this theme, findings related to leadership and management of equipment, supplies and infrastructure were closely linked to the material and structural conditions in the hospitals and district rather than leadership itself being identified as the primary issue or driver. The other three themes directly address leadership styles and practices, describing how strong and supportive leadership can influence maternal health service performance in resource limited hospitals as a primary or direct driver. These themes included: (1) organizational culture, (2) entrepreneurship/innovativeness and (3) accountability.

### Context, infrastructure and working conditions

**Figure 1.**
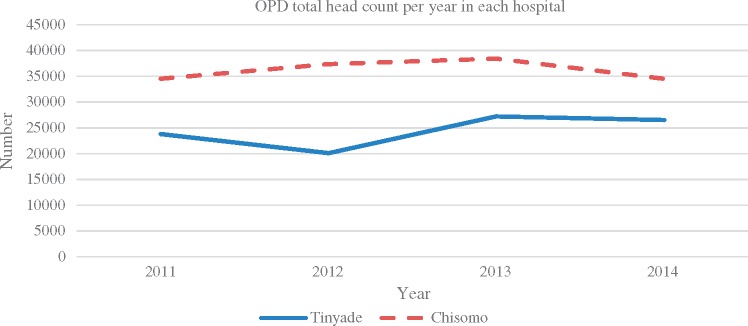
Total number of OPD visits per year at Tinyade and Chisomo hospitals.

**Figure 2.**
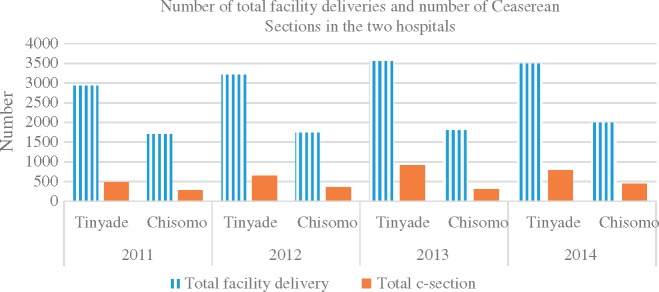
Total number of facility deliveries and caesarean sections at Tinyade and Chisomo hospital.

The two hospitals were similar in terms of external context. Both were once mission hospitals that are now managed by government. Both are situated in a rural poor district and serve similar populations, and they both experience chronic health systems challenges such as under-resourcing, poor supply chains, difficulties to attract and retain new staff, adverse media coverage (particularly on maternal health), low funding levels and unreliable emergency transport. The case mix on the maternity wards was similar in terms of both pattern and severity. The patient load was however different, as illustrated in Figures 1 and 2. Figure 1 shows the Out Patient Department (OPD) headcount per year in the two hospitals, while Figure 2 presents trends in facility deliveries and caesarean sections. Chisomo hospital had more outpatients, by at least a third, compared to Tinyade hospital. One possible explanation reported by study participants was the open door policy that Chisomo had adopted. In contrast to standard practice in South Africa, Chisomo does not turn away any patient, no matter where he or she comes from, even if it is a different district. Respondents reported that the staff believe that if a patient decides to come to their hospital, there must be good reasons and that a patient who has been turned away is unlikely go anywhere else. The Chisomo hospital staff also attributed the high numbers to the relatively good staffing of their hospital departments. Conversely, Tinyade hospital had more deliveries (2500–3500), compared to Chisomo hospital (2000). The percentage of caesarean sections deliveries was similar in the two facilities, except 2013 when Tinyade had more caesarean sections (Table 1).

**Table 1.** Percentage of caesarean sections out of total facility deliveries per year in Chisomo and Tinyade hospital

Years	Chisomo (%)	Tinyade (%)
2011	16.4	16.3
2012	20.3	20.0
2013	16.8	25.4
2014	22.1	22.6

### Equipment and drugs

Table 2 presents data on equipment, tools and drugs and supplies^1^ relative to the National Department of Health (NDOH) recommendations for district hospitals. The data show that the two hospitals had similar equipment available; however, Tinyade had more non-functional items compared to Chisomo. Maintenance of basic equipment was a challenge at both hospitals. Tinyade had deficiencies in vital equipment such as a ventilator, BP machine and thermometer, which are essential to monitor pregnant women for early detection of danger signs. Chisomo hospital had appointed one staff member to coordinate the procurement of equipment for all its departments. We observed in the Management meetings that Chisomo staff were pro-active and reported stock levels were getting low and needed replenishment, which was not the case at Tinyade. Chisomo hospital used daily phone calls to the district as a strategy to quicken the delivery of their stock, and this also enhanced accountability. At times, they used funds they had raised from other sources to repair and maintain their equipment, whereas Tinyade hospital used the government unit (Department of Public Works) for maintenance of equipment; the response to requests was often delayed or there was no response at all.

**Table 2.** Availability and functionality of equipment, tools and supplies at Tinyade and Chisomo hospitals

	Total items assessed	Number of items available and functional	Number of items available but not functional	Number of item not available
Tinyade	75	64	7	4
Chisomo	75	71	1	3

Despite both hospitals having broadly similar resources, Tinyade hospital struggled to improve maternal and perinatal health outcomes. The perinatal mortality rate for Tinyade was 27.9 (per 1000 births over 1000 g) in 2008 and 25.2 in 2015. Chisomo hospital on the other hand, reduced their perinatal mortality rate from 24.6 in 2008 to 17.7 in 2015.

### Human resources and staff management

Hospital managers interviewed at Tinyade hospital complained of understaffing in the maternity section and that staff worked under pressure. This affected their ability to compile patient records and registers, thereby affecting availability of vital information for decision-making. While Chisomo hospital also complained of understaffing, their situation was much better compared to Tinyade, Table 3). Reasons reported for the shortage of staff at Tinyade included high staff turnover and difficulty attracting new staff. Chisomo hospital, on the other hand, reported that staff were provided free accommodation within the hospital premises, which reduced staff travel time and acts as a retention strategy. In addition, the supportive and accommodative leadership style was cited by participants at Chisomo hospital as one of the reasons for their long stay at the hospital:

**Table 3.** Human resources availability and Tinyade and Chisomo hospitals by cadre

Cadre of staff	Tinyade	Chisomo
Advanced mid-wives	3	6
Mid-wives	7	11
Enrolled nurse	7	12
Community health worker	0	0
Social workers	1	1
Nursing assistant	4	7
Full time medical officers	2	9
Other—data capturer	2	2
Other—clinical associates		1
Medical officers on community service	2	2

*‘I was told by a friend how they work here and I couldn’t believe it up until I joined the team. I love it here, you are given space to shine … the managers are very supportive, you have meetings where you discuss your challenges, you feel like you are not alone, there is someone to help you when things gets difficult and too messy’. (Nurse, Chisomo hospital)*

And another respondent said

*‘The management here are friendly, they don’t shout at you and they don’t blame you but help you when you make a mistake as compared to my previous facility, litigation cases!, yhoo!!, you are on your own …’ (Nurse, Chisomo hospital)*

### Client complaints

**Figure 3.**
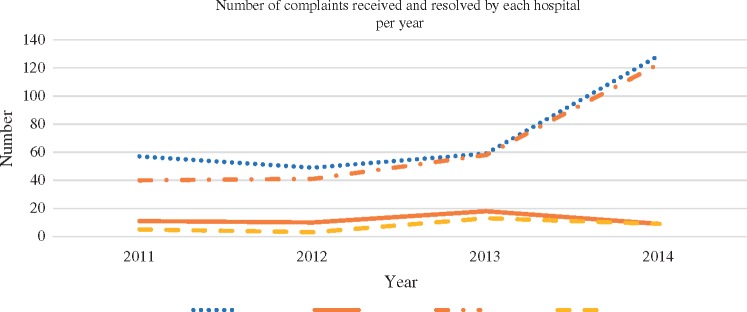
Complaints received and resolved at Tinyade and Chisomo hospitals by year.

Chisomo hospital had fewer complaints from clients in their facility compared to Tinyade hospital. Figure 3 shows that Tinyade hospital consistently had high numbers of complaints from its clients and the trend was getting worse, while Chisomo hospital received very few complaints from its clients. This is one indicator of effective leadership in Chisomo hospital potentially indicating that staff members are responsive to patient needs and therefore patients have less complaints.

### Leadership style and organizational culture

Health staff interviewed at Chisomo hospital described their leadership as supportive, friendly, approachable but ‘firm’. The situation was different at Tinyade hospital, they described the leadership as authoritarian and were feared by their colleagues. At Chisomo, there were more frequent and supportive supervision ward visits by senior staff, and all staff categories were invited to meetings, including support staff like cleaners. While in Tinyade supervision was not frequent and not perceived as supportive, in the sense that challenges and possible solutions were usually not collectively discussed. Most meetings were attended by the facility clinical staff leaders, but not by support staff (e.g. cleaners, data capturers). The staff also complained that senior staff were rarely present in their offices, not used to holding meetings with them, rarely delegating work and poor communicators. At Tinyade, scheduled meetings were often cancelled, unlike at Chisomo where scheduled meetings always went ahead. Respondents gave the example of the *ad hoc* meetings that were organized to address emergency issues, e.g. litigation cases, visitors from the province and national offices. Participants at Tinyade recalled that since the beginning of the year (2015), they only managed to hold 40% of their scheduled meetings with the senior management team, and none with the junior staff, but they had their own as a maternal health unit team. They reported a virtual absence of consultation. Participants in Tinyade also complained of lack of transparency in managing resources, abuse of authority and lack of accountability. This demotivated staff resulting in less commitment to the provision of maternal health services.

*‘Leadership is critical. Leadership is a problem in here. Our leaders need to be trained on how to lead and make things work better, our facility is well known for poor outcomes, what are the leaders doing about this…the outcomes are still bad’.*

Staff at both facilities, however, reported being affected by the overall organizational culture of the National and Provincial Department of Health, which was seen as not respecting the needs of lower level government structures, such as health facilities like theirs. Interviewees complained about the frequency of *ad hoc* meetings that were organized without prior notice. Most of these meetings occurred when they had visiting managers from various programmes, such as HIV/AIDS, Maternal and Child Health, Quality Assurance, National Health Insurance and Nutrition, coming from the National or Provincial Department of Health. There were additional meetings away from the hospitals organized by NGOs, universities and other institutions. This resulted in leadership spending time away from the offices as they were undertaking frequent trips to provincial and district offices. One nurse from Tinyade hospital lamented:

*‘Our Managers are always away travelling to meetings; today they are in Bisho, tomorrow they are in East London, the next day they are in Umtata, it’s like that every week. Our meetings are at times cancelled because of that. Our managers rarely spend a full week here’. (Nurse, Tinyade hospital)*

Table 4 summarizes observations made during perinatal and management meetings on how managers conducted their meetings, engaged with the issues being raised and how staff members participated in the discussions. These experiences have implications for performance of the hospitals given that meetings are to create space for reflection on issues that need to be addressed to improve service performance.

**Table 4.** Conduct of meetings at Chisomo and Tinyade hospital

Perinatal meetings	
Chisomo hospital	Tinyade hospital
Perinatal meetings are supposed to be for hospital only but have taken the initiative to invite colleagues from clinics in their catchment areas and use the platform to discuss cases that need to be dealt with at CHC/Primary Health Care (PHC) level	The sub-district organises the Joint Perinatal meetings with staff from all clinics in the sub-district, operational managers, MOU staff and area managers; and these are held at Tiyande hospital
They had good attendance of staff from their hospital and had a few participants from the clinics. The same group of participants kept on coming back and they said they were motivated by the ‘education sessions’ they had on each case. They referred to them as refresher sessions	The attendance was very poor and the management said it was because of transport and human resources constraints for most clinics. An average of two clinics attended each meeting. They did not have in-service training and had external training that was done by NGOs
Analysed data and presented to the group. Used the reports to track performance, identify problem areas and discussed them in the meetings e.g. indicators that are going down, they used this information to improve maternal and child health outcomes	Reports were presented but they had minimal discussion on the issues raised
	They did not use their data to monitor performance
The perinatal report is sent via emails to all members before-hand for them to read and prepare but some staff do not read the report (some staff by then were not active users of emailing system)	Perinatal reports were distributed just before the meetings
The clinical manager and heads of units prepared and presented the reports and discussed the indicators	The reports were prepared by the information manager together with heads of units, who also presented the reports
Report on adverse events e.g. near miss or deaths—provide full description/account of what happened to discuss and learn from	They also reported on adverse events but without a detailed account of what happened and the lessons learnt. In follow-up meetings there was no clear plan of follow-up of previous discussions
They discussed each case in small groups. Analysed it and discussed what could be done differently, lessons learnt and report back. This facilitated participation of all members and peer learning	Report on adverse events were shared and discussed in the big group but the level of participation was low, only doctors were contributing and there was no conclusion on what should be done differently in future. There was limited participation of nurses and other staff
Minutes were used as a tracking tool, to monitor implementation of what is discussed in meetings	Minutes had no action plans no discussion for the way forward, no action and responsible person section in their minutes
They implemented the recommendations that were feasible to implement at hospital level	The recommendations that were made were not implemented
Other clinics are followed up individually to assess perinatal issues through supportive supervision	There is no follow-up systems reportedly due to lack of resources
The adverse cases were discussed and resolved in groups. They used data to monitor the maternal health outcomes. The graphs they developed were displayed in the Clinical manager and the Maternal Health Unit reception desk	There was fault finding and blame game and there. The PPIP data were not analysed at hospital level and therefore not used to improve program planning
Management meetings	
They had weekly Management meetings where they gave update reports on what was going on in each department, discuss challenges and possible options to address the problems	They had monthly meetings where they gave updates of what was going on in their departments and they also discussed challenges and made good recommendations but those recommendations were not implemented
They used the meeting minutes/record to monitor implementation of what is agreed on in the meetings. Everybody who was assigned to implement the action plans were asked to report back and account	They did not include action plans in their minutes and did not use the minutes to monitor implementation of recommendations

CHC, community health centre; MOU, midwife obstetric units; PHC, primary health care; PPIP, perinatal problem identification programme.

In Chisomo participants noted that rather than blaming and condemning, team members used a collective approach to finding organizational solutions. They acknowledged that learning was a core attribute of their professional development and leadership responsibilities. This viewpoint included openness to learning from mistakes and learning from both your subordinates and peers, with a view that learning is a continuous process. One participant in Chisomo hospital described this practice:

*‘We get together in our management meetings and reflect on what happened, where we made a mistake; we are all accountable, both leaders and operational staff, we have to see what we did and together find a solution’.*

Another participant shared his perspective on developing his leadership skills through daily interactions, with a view that learning is a continuous process.

*‘As a leader you learn new leadership skills each day as you deal with difficult individuals and teams, so the learning never stops. It never stops …’.*

Staff at Tinyade, tended to have negative attitudes towards, and did not trust their leaders. They were more critical and blamed the leadership for most of the things that were not functioning well in the hospital. They for instance blamed the leadership for adverse maternal health outcomes and the increase in the number the number of litigation cases related to maternal health. One nurse said:

*‘Our managers don’t care, we have been complaining about EMS and nothing happened … the ambulances come late and at times never come and the women die while waiting here and managers do not do anything, two women died last month… We used to have at least 1 litigation case per month now; we are receiving new cases almost everyday and the leaders are not taking any action’*

Another nurse said

*‘We were in a meeting in the district, they told us that our neonatal deaths are increasing, it is now around 20+ babies per 1000 births, we were less than that last year, but the managers are not helping us at all to improve, we are on our own …’*

Participants from both hospitals acknowledged the need and value of respecting other people’s views and learning from others with highly diverse backgrounds and experience.

*‘Even if you are the boss, you should still work with everybody, be a leader, yes, but work … . Be open and be willing to learn from others, that is my advice to leaders’.*

Aspects of organizational culture and leadership style are presented in Table 5.

**Table 5.** Other key findings

Theme	Chisomo hospital	Tinyade hospital
Emergency transport	Dedicated obstetric ambulances were more accessible e.g. call an ambulance and get it in an average of 30 min. Could get ambulances from hospital to feeder clinics. There had an EMS depot close to them	Difficult to access dedicated obstetric ambulances transport, turnaround time is long, it ranged between 40 min and 4 h
Information system	Some of the registers were up to date. Few blank places in registers and partograph	Incomplete data (registers, partograph), with a lot of blank spaces
	Data analysed at hospital level, developed graphs and used to inform hospital management decisions at both management and perinatal meetings.	Data were analysed at sub-district level and not shared with end users, it was submitted to the district office
	Presented updates of selected indicators at the PPIP meetings	Presented Perinatal Reports at perinatal meetings and submitted reports to the sub-district and the district offices
	Data were analysed by hospital Managers, but junior staff were not involved—a lost opportunity for capacity building on the same	
Department meetings	Conduct unit meetings to discuss departmental challenges and write reports for presentation to the management meetings	The Maternal Health Unit had their update meetings and also produced reports they submitted to the facility managers
Working relationship—leadership style	Distributed leadership and lots of team work. Worked together in teams in each departments and across teams through management meetings	Hierarchical and there was lack of teamwork, mistrust and lack of feedback
External supervision	Receive a lot of visitors from different programmes (HAST, MCH, Quality Assurance, NHI, Nutrition, Clinical Managers etc.). Complained about high volumes	Also complained about high volumes of visitors from programme managers from the district, province, national, NGOs, researchers
	Comparatively fewer litigation cases	Increase of Litigation Cases—resulted in the increase of visitors from province investigating cases and lawyers representing clients
Infrastructure and equipment pro-activeness	Leadership would go out of their way to either take their equipment for repair or get somebody to repair, at times subcontracted private contractors and the government would pay later but have challenges of late payments	Complained that they are not given maintenance budget, so they were not maintaining their infrastructure and equipment well, e.g. observed loose door handle, blood pressure machines not working because of lack of batteries
	Use hospital board members to help-directly seek help from the Provincial head of the Member the Executive Council for Health (MEC)	Just wait, don’t take initiative
		At times break equipment because of lack of training on how to use it
	Got good staff accommodation	Struggle with staff accommodation
Hospital boards	Functional and support the facility management	Set up on paper, not functional, had not yet met
	Good linkage between facility and the community, have community feedback meetings through the traditional leadership and ward councillors	
	Have representatives from both the business community, youth, traditional levels	
	Had scheduled meetings every quarter	

EMS, emergency medical services; HAST, HIV/AIDS STI and TB; MCH, maternal and child health; NHI, national health insurance.

### Nurturing diversity and teamwork

Closely related to the broad theme of organizational culture was that of nurturing teamwork among diverse individuals. This theme was reported, and elaborated upon, by both managers and staff at Chisomo hospital. Managers at Chisomo hospital reflected that they try to assemble and manage diverse teams with complementary skills and to invest human resources where needed. They acknowledged that ‘no individual was an ‘all-rounder’ and they at times teamed people together to complement their skills. One of the managers shared the following example:

*‘People are not the same, yes, and we acknowledge that, for instance we have got young and energetic doctors here who are always willing to volunteer to help, we’ll give people like that coordinative roles, we have got one elderly nurse who is quiet, respected and a good negotiator, she works well with the community, she has good relationships with them, and we usually send her out together with one of the ‘most talkative staff members’ in the facility, they go together to the health promotions meetings in the community’.*

As illustrated in the above example the elderly nurse was the key person who was given the responsibility of negotiating access, organizing meetings and the ‘talkative staff member’ was given the responsibility to deliver campaign messages in the community meetings, and therefore managers made use of, and appreciated the value of diversity in the team.

At Chisomo, categories of staff worked together as a team in a range of regular meetings to discuss management issues (Management meetings), clinical issues (Clinical governance meetings) and maternal and child health (peri-natal and paediatric morbidity-mortality meetings).

*‘Whatever problem we have, we usually present it in one of these meetings and ask for suggestions, and people come up with the brilliant ideas. They are happy when you use their ideas and acknowledge them’.*

Other participants echoed similar sentiments that participating in meetings where they discussed the challenges at Chisomo hospital and discussing possible solutions motivated them to implement the suggested strategies and built a sense of responsibility. They said they *‘*feel being part of a team’. They emphasized the importance of building and sustaining relationships in managing and providing care in complex and resource-limited environments. The leadership used strategies like reaching out and engaging all the key partners working in maternal health in their catchment areas, open and continuous communication with an emphasis on listening. The contributions of others were valued and were used to improve services and as a means to gain support for the implementation of any new initiative.

*‘We listen to everybody’s opinion and suggestions, we actual get good points from the meetings, and we make use of them. I think people like it, because they will usual support what they suggest …’ (Manager, Chisomo hospital)*

Investing in relationships by empowering others to voice their opinions and contributions was perceived as important by participants who saw it as a benefit in terms of nurturing trustworthiness, confidence and support among staff in all the departments who could work with them in the implementation of future initiatives. A Chisomo manager explained:

*‘You let people speak … if you give people an opportunity to speak, they will surprise you, they might even know more than you do, they have information that can help you … and if you take what they say serious and they see you using their advice, you find them more trusting and become easy to work with’.*

The situation was different in Tinyade. There were pockets of teams in different units. Staff in the maternal health unit for example said they had their own unit meetings as a team but they felt isolated from the rest of the hospital. None of them was a member of the management committees.

One midwife observed

*‘We have not had meetings where we discuss our challenges as a team, maybe senior managers do but we don’t. We are on our own here, we meet as a unit’*

### Entrepreneurship and innovativeness

Both facilities complained about the poor procurement systems and experienced delays in receiving their orders and at times received wrong orders. One of the strategies Chisomo hospital leadership used was to by-pass the hierarchical structures, at both district and provincial levels, to access certain services, e.g. order drugs or equipment, or to recruit staff. They said at times they contacted the NDOH or Provincial offices directly to obtain certain products e.g. unfreeze posts, or replace broken equipment. They used their networks, such as the hospital board, to get things done because the normal bureaucratic channels were at times ineffective. This helped them speed up procurement processes. Other innovative strategies used by both facilities to cope with shortage of drugs included borrowing from other health facilities asking for assistance from NGOs, or asking their procurement officer to make several calls to follow-up.

Our data show that the managers in Chisomo hospital used their entrepreneurial skills and other innovative ways to ensure the effective running of the hospital. For example, they set up a community NGO that was used to support their community health projects, raise funds for the hospital and create a website. Another example is that at times they used their own resources to buy some of the urgently needed products such as batteries for blood pressure machines and they would claim reimbursement afterwards. These were personal initiatives which demonstrate pro-activeness to ensure the functioning of hospital services. Another example they offered was assistance they received in recruiting/sourcing additional human resources like doctors from overseas:

*‘We welcome every partner who wants to work with us, we discuss our challenges and our priority areas. We approach some and ask for assistance … for instance we had a malnutrition problem and deliberately approached them and asked for help. We even have a website where we share information about ourselves’*

Even though the implementation of national policy on hospital boards and clinic committees was launched in 2013, Tinyade hospital is still struggling to set up its hospital board and make it functional. Chisomo hospital managers were innovative and proactive in the provision of transport to the Board meetings, facilitated the development of action plans and actively involved them in discussing some of the challenges the facility had and using them to ‘canvas/lobby for resources from the provinces’. One hospital board member said:

*‘The facility here keeps us informed of their challenges … we also help where we can, I have even gone to the MEC to ask him and national to lift up the freeze of some of our posts, and even to support the building of new infrastructure. This hospital is old’.*

### Accountability

Both hospitals conducted regular perinatal meetings to discuss adverse cases (e.g. near miss, maternal deaths) to identify systems failures and accountability for poor outcomes. Chisomo also used perinatal meetings as a learning platform. We observed that they discussed one adverse case in each meeting, and they worked in groups (a mix of doctors and nurses) to discuss mistakes and lessons learnt. Feedback sessions also included lectures on specific topics (e.g. diagnosis, management and referral of hypertension or breech babies). So they learnt from both their peers and the senior clinicians in the group. Instead of blaming individuals, the participants used a collective approach to accountability to find solutions focusing on a systems approach. One participant described:

‘The good thing is that all of us become responsible for any adverse outcome, not just one individual … and we use the perinatal meetings to learn from mistakes and for me they have become refresher course sessions, for example the session we did today, management of breech babies, I knew some of the stuff but I was getting rusty, you understand what I mean, I got good tips from colleagues today …’

To ensure accountability, Chisomo hospital used minutes from the meetings as a tool for monitoring performance. In their minutes they recorded actions to be taken and the person responsible. In their management meetings each head of unit gave an update and accounted for the action points from the previous minutes. However, Tinyade hospital did not make use of their perinatal meeting minutes to monitor individual and systems performance, and identify responsibility and accountability for actions which were needed to improve care. We reviewed meeting minutes and observed they had instances where the same items were discussed in > 4 sittings and similar recommendations made, but were not actioned. They had no accountability system in place to assure corrective actions were taken.

In an effort to sustain momentum and inspire others, acknowledgement was made for good performance at Chisomo hospital and those who did not perform well were encouraged and provided suggestions on how they could better respond to a particular challenge. At the end of each meeting they make a plan of action, also taking resource availability into account. In all their meetings they interrogated data to track their performance and identify areas for improvement.

## Discussion

This study shows how leadership influences the function of maternal health services in district hospitals. Study participants described aspects of their leadership experiences. Staff at the better performing facility appreciated the teamwork and collective approach in dealing with challenges, whereas staff at the poorly performing facility were more critical and blamed the leadership for poor performance. These aspects of leadership style, such as use of data to improve service delivery, supportive leadership, teamwork have been reported in other studies (Puoane *et al.* 2008; Curry *et al.* 2012; Lembani *et al.* 2015). Sola *et al.* (2016) study on self-perception of leadership styles and behaviour in primary health care classified this leadership style as ‘transformational’ and explained that it encourages teamwork, collaboration, empathy and the acceptance and use of innovativeness which results in improvement of outcomes as compared to the more rigid and bureaucratic leadership styles. Similar observations were made by other studies on transformational leadership (Schaubroeck *et al.* 2007; Top *et al.* 2013).

There was a strong emphasis on supportive supervision, nurturing relationships and in-service training in the successful hospital. The senior clinical staff, the nurses and doctors natured and supported junior and other staff through their routine management and clinical governance meetings, ward visits and in-service training, and were proactive in introducing new changes in maternal health protocols to their staff. They also made use of their ideas and contributions when addressing service delivery challenges and improving practices in the maternal and children’s ward. In addition to that, there was wider consultation, better supportive supervision and monitoring of maternal and child health outcomes across a variety of meeting forums including perinatal and management meetings. In Lembani and other’s study (2008) accountability, staff motivation, better communication, accommodative and supportive leadership emerged as the key driver of performance in the maternal health programmes. This appears to be particularly true when strategies are implemented in combination and when, as in this case, are focused on routine tasks and problem solving (Puoane *et al.* 2008).

The open discussion of adverse cases in perinatal meetings and in-service training helped other cadres of staff become confident in managing similar cases, and also reinforced knowledge, but also provided space for learning, informal engagements, relationship building and better communication across different cadres of hospital staff. These social aspects created an opportunity for leadership to strengthen their teams and increased likelihood that any action or innovation they agree on as a team will be implemented and sustained (Lembani *et al.* 2015). In contrast, the poorly performing hospital relied on NGO and Provincial Department of Health training, which took them away from the hospital, instead of using their existing structures like the perinatal meetings and in-service training and induction. As a result, many staff did not have adequate skills and knowledge, which negatively affected the functioning of maternal health services and quality of care.

Participants from the better performing Chisomo hospital argued that their leaders were supportive, approachable, friendly and they used a teamwork approach to address challenges and this motivated them to respond to challenges better. Alloubani *et al.* (2014) in a review of effects of leadership styles on quality of services in health care found that transformational leadership characteristics have positive effects on organizational outcomes, including: ‘teamwork success, effectiveness, staff satisfaction, commitment and extra effort and others’. Other studies have also shown that strengthening leadership can increase the chances of improving performance (Lembani *et al.* 2015; Sola *et al.* 2016). Al-Sawai (2013) further emphasized the need for effective leadership to focus on the dynamic relationships between leadership values, culture, capabilities and the organizational context anchored by a high level of self, team and organizational awareness.

Using data, Chisomo staff were able to plan and develop strategies to deal with specific problems, potential risks and challenges in their units as a team. On the contrary, Tinyade had data problems with the quality of their data and lack of data analysis for planning and monitoring of performance for programme improvements. Their data were analysed at sub-district level. This observation was also reported in a health systems resilience study done in the same region; where the quality of data was poor and the staff members were not using it to monitor and improve performance (Lembani *et al.* 2015).

Both facilities reportedly made use of perinatal meetings to discuss adverse cases, monitor and account for poor outcomes, but there was limited use of lessons learnt in Tinyade as compared to Chisomo, and the latter monitored implementation through meeting records. A recent literature review illustrated how accountability mechanisms, such as perinatal and maternal death surveillance reviews, have failed to make health care providers and managers account for the quality of services as implementation rates of recommendations from reviews were low (Hilber *et al.* 2016).

### Limitations

One potential limitation to this study relates to generalizability as it is a descriptive, context specific case study with a small sample. However, understanding one case promotes understanding of similar cases and of general issues related to the phenomenon under study (Thorne *et al.* 2004). It is also difficult to ‘prove’ improvement in the performance of the hospitals to leadership as there were other health systems interventions being implemented, but our data demonstrate that the type of leadership or leadership style played a major role in driving change. The quality of the records at facility level was not good so we were not able to use data for some of the outcome indicators.

Despite these limitations, our study strengths include use of various data collection methods and participants with diverse leadership roles that contributed to understand the complexity of facility level leadership.

### Policy implications

Implications of this study are that interventions to improve the maternal health services are influenced by the contextual environment in which they are implemented, including the prevailing leadership. Strengthening leadership style and practices is, therefore, important for improving the maternal health services and quality of care. South Africa, for example, in particular has placed considerable emphasis in recent years on improving health leadership and has developed several strategies such as ‘the Primary Health Care re-engineering’ and ‘the Ideal clinic’ (Gilson and Diaire 2011). Despite these efforts, inadequate leadership capacity among managers has been identified as a key challenge constraining effective health service delivery (Wilson *et al.* 2015). Other studies have also highlighted the importance of changing the approach that is currently dominant in the maternal health programmes, namely a focus on technocratic requirements, like development of protocols/guidelines and tools (Lembani *et al.* 2015). This research reinforces similar findings in maternal health and other programme areas that, while the technocratic and financial issues (including guidelines and tools) are valuable and essential, their successful application will be determined by the context and, in particular, leadership capacity (Puoane *et al.* 2008). This study shows that strong and committed leadership promotes effective mobilization of teams and provides an environment for improved facility performance, even when resources are limited. Action research through peer support is on-going to assist Tinyade hospital to address challenges described, including leadership styles.

## Conclusion

In much of the literature hospital performance is largely attributed to resource availability and hardware issues, such as finance, human resources, equipment, drugs and supplies; and excludes the software aspects (values, trust, power, interrelationships and others) (The Governance Institute 2015). The leadership roles of those responsible for managing all these resources in the facilities and their impact on the overall performance of their institution are often ignored. This study has demonstrated that strong and committed leadership promotes effective mobilization of resources and teams, and creates the conditions for good performance in districts hospitals, even when they have resource constraints and poor health system.

Clear differences in organizational culture and leadership style emerged between the two hospitals, one with low perinatal mortality rates and one with higher rates. The observed differences that affect the functioning of maternal health services were use of data, supportive supervision, teamwork, in-service training, accountability and feedback systems through regular perinatal and management meetings. Underlying factors were differences in leadership style, including teambuilding, innovation and entrepreneurial skills and opportunities for learning from each other and social interaction that strengthened their relationships. Even though they both had similar infrastructure, in the successful hospital, there was a combination of favourable conditions that provided the opportunity and context within which maternal health services could be improved. In the poorly performing hospital, lack of approachable and effective leadership demotivated staff and resulted in mistrust, frustration and lack of commitment.

Even though the leaders played an important role in influencing change, sustainability of performance in health facilities still needs strong and well-developed systems. Good performance and better maternal health services in Chisomo hospital was driven by a few individuals and might be difficult to sustain if key individuals were to leave, especially in a context of a weak health system as in our study site.

## Footnotes

1 Checked whether they had basic equipment like blood pressure machine, thermometer and whether they were able to do some of the signal functions e.g. (1) Administer parenteral antibiotics; (2) Administer uterotonic drugs (i.e. parenteral oxytocin) and (3) Administer parenteral anticonvulsants for pre- eclampsia and eclampsia (i.e. magnesium sulfate).
